# Tertiary institutions’ social health insurance program (TISHIP) in Southwestern Nigeria: How knowledgeable are the end-users?

**DOI:** 10.1371/journal.pgph.0004545

**Published:** 2025-05-07

**Authors:** Iretioluwa Mary Bamtefa, Ibukunoluwa E. Okunade, Oluranti Babalola, Temitope Ilori, Folashayo I.P. Adeniji, Akinbode S. Fafiolu, Taiwo A. Obembe

**Affiliations:** 1 Department of Health Policy and Management, Faculty of Public Health, College of Medicine, University of Ibadan, Nigeria; 2 Department of Microbiology, College of Medicine, University College Hospital, Ibadan, Nigeria; 3 Department of Social Work, University of South Carolina, Columbia, South Carolina, United States of America; 4 Department of Family Medicine, University College Hospital, Ibadan, Nigeria; 5 Department of Epidemiology, Georgia State University, Atlanta, GeorgiaUnited States of America; School of Public Health, College of Health Science, Addis Ababa University, ETHIOPIA

## Abstract

The Tertiary Institutions Social Health Insurance Program (TISHIP) was created by the National Health Insurance Authority (NHIA) to enhance health insurance coverage among students in tertiary institutions in Nigeria. Despite being in existence for many years, a significant number of eligible students remain uncovered by the program. It is unclear whether the intended users are sufficiently aware of the program’s existence and benefits. Therefore, this study assessed the level of knowledge about the TISHIP program and related factors among students enrolled in selected tertiary institutions in Southwest Nigeria. Using a descriptive cross-sectional study design, 430 students were surveyed using a multi-stage random sampling technique from the University of Ibadan, Ibadan, Nigeria and the Federal College of Agriculture, Ibadan. Data were collected using a structured self-administered questionnaire of twenty-one questions using both a 4-point Likert scale questions and other questions not on a Likert scale to comprehensively assess the students’ knowledge and perceptions. Responses to the Likert scale were reversed, so that “Strongly disagree” had a score of “1”, and “Strongly agree” was assigned a score of “4”. Overall, six questions were used to determine the knowledge of TISHIP, and the highest score was therefore “24”. Persons with scores corresponding to the mean value and above (≥12 points) were categorized to have “Good knowledge of TISHIP”, while those with lower score (<12) were said to have “Poor knowledge of TISHIP”. One question “What is your view about TISHIP” was used to determine respondent’s perception of TISHIP. Responses were arranged as follows: “Good”, “Poor”, and “Not sure”. “Good” and “Not sure” were merged as “Poor”. Chi-square tests were conducted to determine the association between knowledge and utilization of TISHIP with sociodemographic characteristics of the respondents. The level of significance was set at p < 0.05. The mean age of the respondents was 21.2 ± 2.9 years. More than half of the respondents (52.1%) were males, and 76.0% were university students. Overall, 46.0% of the students had good knowledge of TISHIP, and 173 (73.0%) had a good perception of the program. Being male (Chi-square = 0.043, p = 0.836) was associated with having a good knowledge of TISHIP. University students were approximately 3 times more likely to have good knowledge of TISHIP compared to college students (AOR = 2.616, 95%CI = 1.209 – 5.658, p = 0.015). Postgraduate students were three times more likely to have a good perception of TISHIP (AOR = 3.257, 95%CI = 0.622 – 17.051, p = 0.162) than undergraduate students. Knowledge of TISHIP was low while perception of the program was promising among students in tertiary institutions. Increased efforts to effectively communicate existence and benefits of TISHIP will be critical to the success of the program.

## Introduction

Access to quality and affordable healthcare for students, all Nigerians, and legal residents is critical to achieving universal coverage nationwide. It will also facilitate the country’s overall growth. In 2008, the Tertiary Institution Social Health Insurance Program (TISHIP) was created under the National Health Insurance Authority. The target markets for this health plan are tertiary institutions. Students enrolled in universities, colleges of education, polytechnics, schools of nursing and midwifery, and other specialized colleges are eligible to apply for the TISHIP Scheme.

How a country finances its healthcare program is crucial to determining the quality of healthcare delivery in that country. Consumer payment methods can be grouped into pre-service payments and point-of-service payments. The former typically refers to indirect pooled payments in the form of health insurance schemes. The latter applies to out-of-pocket (OOP) payments [1].

The World Health Organization, in 2005, passed a resolution that social health insurance should be one of the strategies used to mobilize more resources for health, risk pooling, increased access to health care for the poor, and the delivery of quality healthcare in all its member states, especially in low-income countries [2].

Most developing countries have recently begun the implementation of some health financing strategies, focusing primarily on social health insurance schemes. This will help to provide effective and efficient health care for their citizens, particularly the poor and vulnerable [3]. These reform programs and strategies were designed to provide easy access to healthcare at an affordable price via various prepayment systems, thereby improving citizens’ overall health status [4].

Like other African countries, Nigeria, enacted the National Health Insurance Scheme Act, 1999, to provide access to qualitative, equitable, and affordable healthcare for all Nigerians [5]. The first phase of this scheme was implemented in 2005, with the formal sector social health insurance program taking the lead [6]. This scheme recognizes the primary beneficiary, their spouse, and four biological children under the age of 18. More dependents are recognized with additional contributions. To cater for the healthcare needs of Nigerians who have not been captured by this scheme, similar initiatives like the Tertiary Institutions’ Social Health Insurance Programme (TISHIP) were introduced.

The Tertiary Institutions’ Social Health Insurance Program (TISHIP) is a Nigeria-based social security system that pays for students’ health care through pooled mandatory contributions made by students and the government. The program promotes students’ health to foster a favourable learning environment and unhindered academic activities. Poor health could hinder academic pursuits [7]. The program provides good health services and protects students from the financial hardships of huge medical bills by ensuring the availability of funds for tertiary institutions’ health centres. It covers students enrolled in universities, colleges of education, polytechnics, schools of nursing and midwifery, and other specialized colleges without bias or discrimination [8]. It is priced at a community-rated sum of NGN2,000 per academic session. This programs’ key stakeholders include NHIA, tertiary institutions, health maintenance organizations (HMOs), healthcare facilities, the Student Union Government (SUG), and tertiary institution regulatory bodies.

Awareness of TISHIP, its acceptance by students, and proper implementation in health care facilities, are critical for the realization of its goals and objectives. Prior studies found that students rarely visited institutions’ health care facilities due to high medical service costs, poor working conditions, and unsatisfactory referral services [8]. TISHIP aims to address these challenges. Studies indicate that health policies are poorly understood, resulting in inadequate knowledge about the policy’s standard guidelines and regulations [9]. Health information sources and the quality of services rendered play a pivotal role in the utilization of health services by Nigerian tertiary students. Understanding how the availability of TISHIP information sources improves undergraduate students’ access to healthcare, aids policymaking and promotes universal healthcare coverage.

Nigeria, the largest country in Africa by population, has a compromised healthcare sector. The health insurance coverage of men and women aged between 15 – 49 is about 3% [10]. Besides, despite the creation of TISHIP, many Nigerian tertiary students are not covered by the scheme. Inadequate information dissemination and poor accessibility to quality health care are some of the problems they experienced. Institutional information sources on policy, objectives, coverage, and programs of the TISHIP include television, radio, orientation programs, library resources, seminars, lecturers, peer groups, billboards, handbills, and the students’ handbooks. Existing literature suggests that the use of certain information sources varies in degree among different socio-economic and demographic groups [11]. The program’s implementation by healthcare professionals and the students’ understanding of its establishment play a role in its success.

The importance of healthcare services cannot be overemphasized. A lack of access to it constitutes a barrier to healthcare delivery in Nigeria. Those engaged in the program need more understanding and awareness of the health insurance operations. Complaints have been filed alleging that providers denied enrolees their full entitlements and that some providers demanded extra costs. They claimed that the service requested was not included in the benefit package. Insured individuals have expressed their dissatisfaction with the approach and conduct of social health insurance program service providers, the lengthy wait times, and poor service quality. Undoubtedly, issues relating to the delivery of health services should be understood and addressed [12].

Following the creation of the TISHIP by the Nigerian government, inadequate information dissemination and misinterpretation of the insurance scheme persist. Years after the enactment of the National Health Insurance Authority (NHIA) in Nigeria, there is still a low coverage rate and slow progress in health insurance. This is attributable to inadequate knowledge and a poor enrollee-provider relationship [6].

Students among the dependent population may fall ill while in school. This, if not promptly and appropriately managed, will negatively affect their academic performance. Healthcare is an enormous financial burden on students and their guardians [13].

Access to healthcare for all students is possible only if they have information about the services available to them and their attendant benefits. The slogan ‘health is wealth’ captures the importance of being healthy. The state of healthcare services in Nigeria falls short of the average citizen’s expectations. A study [14] revealed that the provision of TISHIP as part of health care services could be of great help to students. It seeks to ensure that the occurrence of an unexpected event does not place an individual under undue stress.

This study, therefore, assessed the perception and knowledge of tertiary students of TISHIP in Ibadan, Nigeria. Hence, the findings and recommendations will be useful in the management of NHIA as an agency of the Federal Government of Nigeria, the students of research institutions, and policymakers.

## Methods

### Study design

The study employed a descriptive cross-sectional study design to assess the overall knowledge, perception and factors associated with the enrolment of undergraduate students in health insurance using the tertiary institution social health insurance program (TISHIP) in Ibadan, Southwestern Nigeria.

### Study population

The study was conducted at two selected tertiary institutions: the University of Ibadan and the Federal College of Agriculture, Ibadan. The institutions were purposefully chosen, as both are classified as tertiary institutions, and tertiary institutions are not limited to universities alone. The University of Ibadan is the first University in Nigeria. Originally known, as a college of the University of London under a special relationship scheme, became a full-fledged independent university in 1962. There are 16 faculties in the University of Ibadan (University of Ibadan, 2021). The Federal College of Agriculture, Ibadan (previously known as the School of Agriculture) was founded in 1921. It is Nigeria’s and West Africa’s first agricultural institution.

### Sampling technique

The multi-stage sampling technique was employed for the recruitment of study participants. First, the University of Ibadan and the Federal College of Agriculture Ibadan were purposively selected on the criteria that tertiary institutions are not limited to universities alone. Most research is conducted there, and many research participants and respondents were chosen from there [15]. In each institution, faculties/departments were randomly selected by 50% balloting: 8 out of the 16 faculties at the University of Ibadan, Ibadan, and five out of the nine departments at the Federal College of Agriculture, Ibadan. Using proportional sampling, each faculty/department selected was allocated a calculated proportion of the sample size. The selection process was finally conducted at faculty/department lecture theatres.

### Sample size estimation

Using Leslie Kish formula calculation technique, a calculated minimum sample size of 385 at a 5% level of significance. A prevalence of correct knowledge of TISHIP (47.6%) amongst undergraduate students was used as our reference value [13]. This was increased to 430 to adjust for a 10% non-response.

### Data collection

Data were collected using self-administered questionnaires, which included participants’ socio-demographic information, knowledge of TISHIP, and perception of TISHIP between 28 April 2022 and 27 April 2023. Pre-test of the questionnaires was carried out on 10% of the sample size (43 students) in a similar tertiary institution. Reliability testing of the scales yielded an overall Cronbach’s Alpha of 0.83.

### Data analysis

A questionnaire comprising of twenty-one questions was used to assess students’ knowledge and perception of TISHIP. The questionnaire consisted of both a 4-point Likert scale questions (ranging from Strongly Agree to Strongly Disagree) and other non-Likert scale questions to comprehensively assess the students’ knowledge and perceptions. Responses to the Likert scale were categorised such that “Strongly Disagree” and “Disagree” were considered incorrect answers and assigned a score of “0”, while “Strongly Agree” and “Agree” were considered correct answers and assigned a score of “1”. Overall, six (6) Likert scale questions and four (4) non-Likert scale questions were used to assess knowledge of TISHIP. Each correct answer from the non-Likert scale questions was awarded 1 point. The total possible score was 10 points (6 points from the Likert scale questions and 4 points from the non-Likert questions. Participants were categorised into two groups of Good Knowledge of TISHIP (with 5 points or higher) and Poor Knowledge of TISHIP (with less than 5 points). One question “What is your view about TISHIP” was used to assess the respondent’s perception of TISHIP. Responses were categorised as follows: “Good”, “Poor”, and “Not sure”. “Good” was considered a positive perception, “Poor” was considered a negative perception, and “Not sure” was considered a neutral perception, “Poor” and “Not sure” were merged as “Poor”. Chi-square tests were conducted to determine the association between knowledge and perception of TISHIP with sociodemographic characteristics of the respondents. The level of significance was set at p < 0.05 [13].

### Ethical consideration

The study approval was obtained from the University of Ibadan/University College Hospital ethical committee on registration number UI/EC/22/0067. After a thorough explanation of the research, participants’ written informed consent was obtained to conduct the survey. The principles of research ethics were put into practice. They are non-maleficence, beneficence, justice, and respect for autonomy [16]. All data obtained during the study was treated with confidentiality and anonymity. Data access was restricted to the two research assistants responsible for the data collection, the data analyst, and the principal investigator.

## Results

### Socio-demographic characteristics of participants

A total of 430 male and female students were surveyed using the questionnaires. Three hundred and twenty-seven (76.0%) were University of Ibadan students, while the remaining 103 (24.0%) were Federal College of Agriculture students. The mean age of the respondents was 21.2 ± 2.9 years. One hundred and eighty-six students were aged 20–22 years; 224 (52.1%) were males, and 301 (70.0%) practiced Christianity (Table 1).

**Table 1:** Socio-demographic characteristics of the respondents.

Variables	Frequency (n) N = 430	Percentage (%)
**Age (Years)**		
≤ 19	132	30.7
20-22	186	43.3
≥ 23	112	26.0
**Sex**		
Male	224	52.1
Female	206	47.9
**Religion**		
Christianity	301	70.0
Islam	129	30.0
**Marital status**		
Single	421	97.9
Married	9	2.1
**Level**		
Undergraduates	407	94.7
Postgraduates	23	5.3
**Faculty**		
Science and Technology	121	28.1
Agriculture and Forestry	104	24.2
Sociology	35	8.1
Education	63	14.7
Pharmacy	35	8.1
Arts	72	16.7
**Institution type**		
University	327	76.0
College	103	24.0

### Knowledge of TISHIP

All the respondents were aware of the National Health Insurance Scheme. Overall, 237 (55.1%) had ever heard of TISHIP, 114 (48.1%) strongly agreed that TISHIP was a subsidiary program under NHIA, 51 (21.5%) strongly agreed that students and the government jointly funded TISHIP, and 55 (23.2%) strongly agreed that TISHIP ensured that every student in higher institutions had access to quality health care. Furthermore, 96 (40.5%) strongly agreed that TISHIP protected parents and guardians from the financial hardships of huge medical bills. Other characteristics related to knowledge of TISHIP among respondents are shown in Table 2.

**Table 2:** Knowledge of TISHIP among study participants.

Characteristics	Frequency (n)	Percentage (%)
**Ever heard of TISHIP (N = 430)**		
Yes	237	55.1
No	193	44.9
**Meaning of TISHIP (n = 237)**		
Tertiary Institutions’ Social Health Insurance Programme	217	50.5
Today’s Institution Students Health Insurance Programme	3	0.7
Today’s Institutions Social Health Insurance Programme	7	1.6
Tertiary Insurance Students Health Institutions Programme	10	2.3
**TISHIP is a subsidiary program under the NHIA (n = 237)**		
Strongly Agree	114	48.1
Agree	105	44.3
Disagree	7	3.0
Strongly Disagree	11	4.6
**Student and government’s contributions are involved in funding TISHIP(n = 237)**		
Strongly Agree	51	21.5
Agree	145	61.2
Disagree	34	14.3
Strongly Disagree	7	3.0
**TISHIP promotes improvement in healthcare delivery in Nigeria (n = 237)**		
Strongly Agree	76	32.1
Agree	119	50.2
Disagree	22	9.3
Strongly Disagree	20	8.4
**TISHIP ensures that every student in higher institution has access to quality health care (n = 237)**		
Strongly Agree	55	23.2
Agree	149	62.9
Disagree	28	11.8
Strongly Disagree	5	2.1
**TISHIP ensures that parents and guardians are protected from the financial hardship of huge medical bills (n = 237)**		
Strongly Agree	96	40.5
Agree	116	48.9
Disagree	20	8.4
Strongly Disagree	5	2.1

### Knowledge of TISHIP

**Fig 1:**
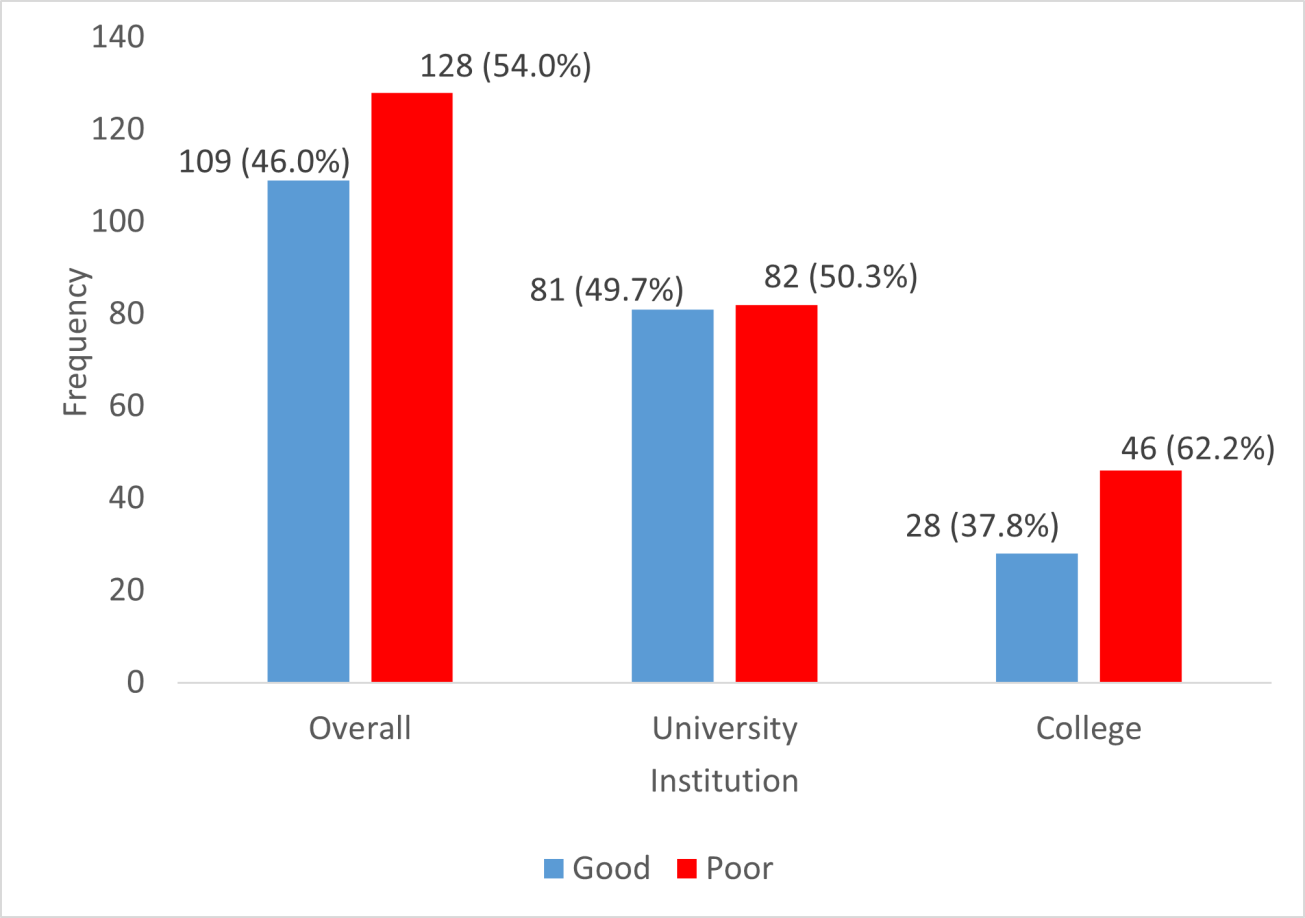
Knowledge of TISHIP among the respondents.

A total of one hundred and nine (109) participants, representing 46.0%, had good knowledge of TISHIP. Eighty-one (81) university students, representing 49.7%, had good knowledge of TISHIP, while 28 (37.8%) college students had good knowledge of TISHIP (Fig 1).

### Sources of information on TISHIP

**Fig 2:**
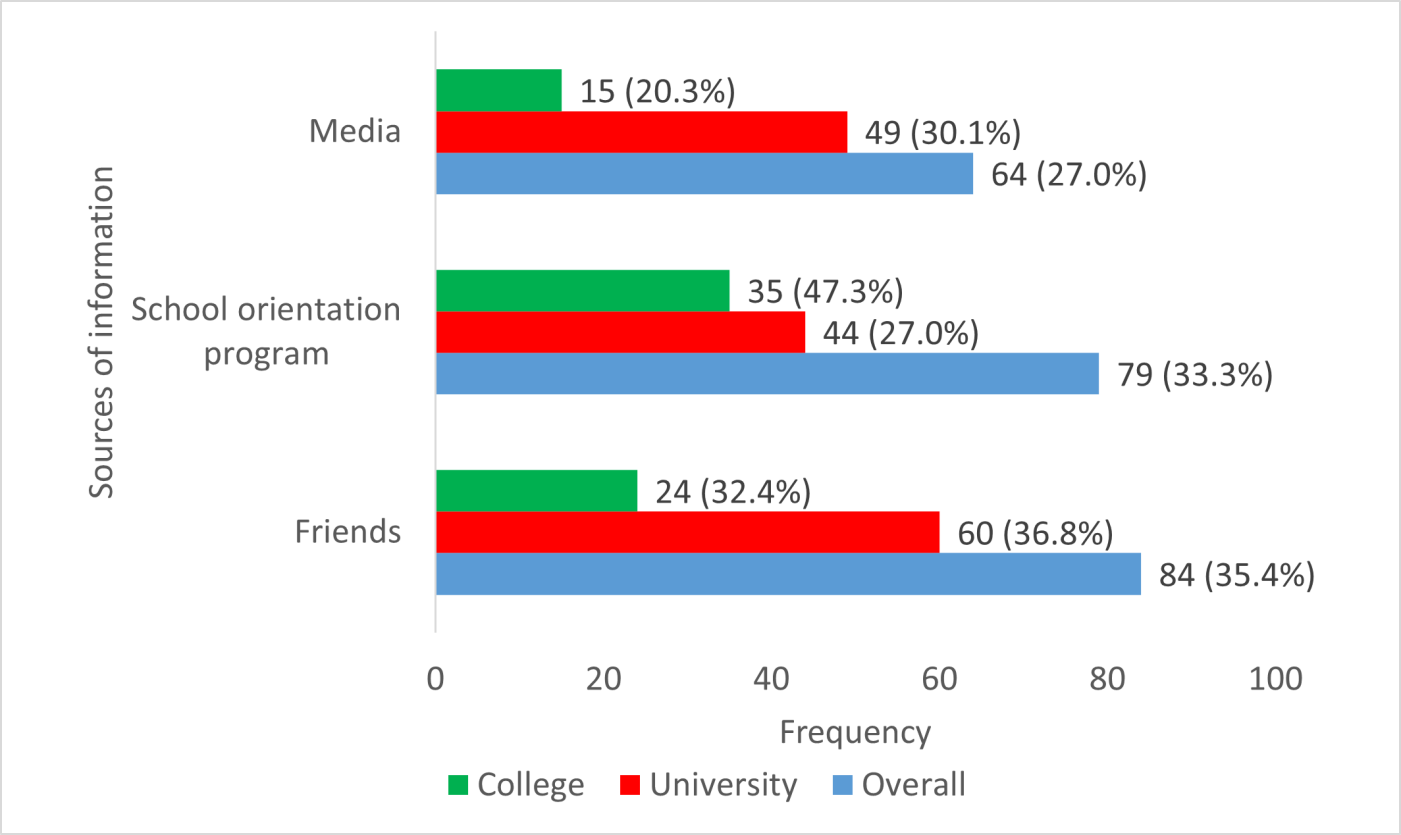
Sources of information on TISHIP among university and college students.

Eighty-four (84) students, representing 35.4%, gained information about TISHIP from their friends, and 79 (33.3%) acquired knowledge of TISHIP from school orientation programs. Sixty (36.8%) university students gained information about TISHIP from their friends, compared to 24 (32.4%) college students who gained information from the same source. Forty-four (27.0%) university students acquired knowledge of TISHIP from school orientation programs, compared to 35 (47.3%) college students who acquired knowledge from the same source. Other sources of information on TISHIP are shown in Fig 2.

### Perception of TISHIP

**Fig 3:**
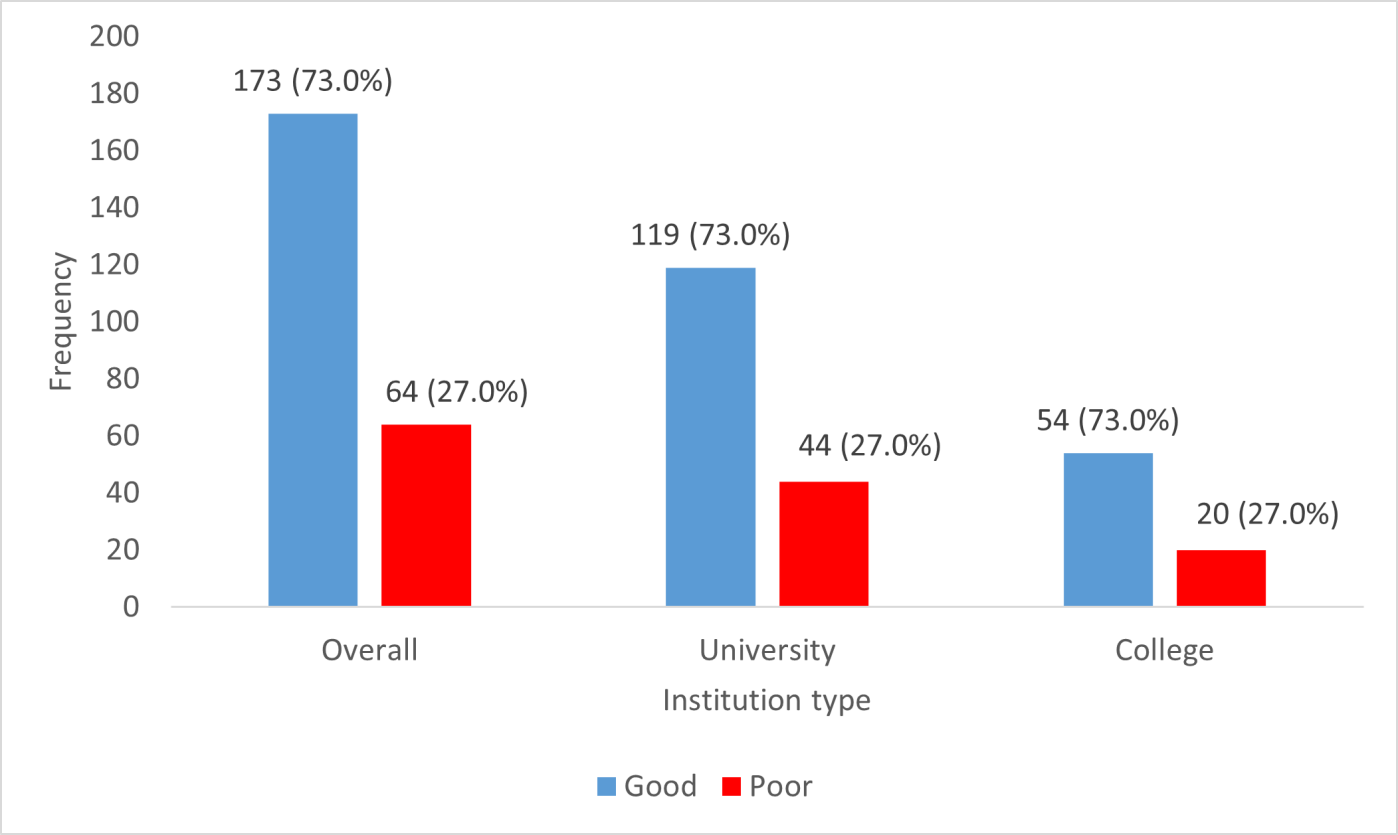
Perception of TISHIP among students enrolled in selected tertiary institutions in Ibadan, Oyo State.

One hundred and seventy-three (173) students, representing 73.0%, had a good perception of TISHIP. Among them, 119 (73.0%) were university students, while 54 (73.0%) were college students (Fig 3).

### Factors associated with knowledge of TISHIP

Thirty-three (33) students, representing 42.9%, who were aged 19 years and below, had good knowledge of TISHIP compared to 40 (47.1%) who were aged 20–22 years and 36 (48.0%) who were aged 23 years and above (Chi-square = 0.465, p = 0.792). Also, 59 (45.4%) male students had good knowledge of TISHIP compared to 50 (46.7%) females (Chi-square = 0.043, p = 0.836). Also, 106 (48.0%) undergraduate students had good knowledge of TISHIP compared to 3 (18.8%) postgraduate students (Chi-square = 5.126, p = 0.024) (Table 3).

**Table 3:** Association between knowledge of TISHIP and socio-demographic characteristics of respondents.

	University		College		Total	
Variables	Knowledge of TISHIP		Knowledge of TISHIP		Knowledge of TISHIP	
	Good n (%)	Poor n (%)	Good n (%)	Poor n (%)	Good n (%)	Poor n (%)
**Age (Years)**						
≤ 19	4 (25.0)	12 (75.0)	7 (58.3)	5 (41.7)	33 (42.9)	44 (57.1)
20-22	13 (48.1)	14 (51.9)	0 (0.0)	9 (100.0)	40 (47.1)	45 (52.9)
≥ 23	11 (42.3)	15 (57.7)	3 (75.0)	1 (25.0)	36 (48.0)	39 (52.0)
	χ^2^ = 2.284, p = 0.319		χ^2^ = 9.722, p = 0.002^*^		χ^2^ = 0.465, p = 0.792	
**Sex**						
Male	17 (43.6)	22 (56.4)	6 (37.5)	10 (62.5)	59 (45.4)	71 (54.6)
Female	11 (36.7)	19 (63.3)	4 (44.4)	5 (55.6)	50 (46.7)	57 (53.3)
	χ^2^ = 0.337, p = 0.562		χ^2^ = 0.116, p = 0.998		χ^2^ = 0.043, p = 0.836	
**Religion**						
Christianity	19 (40.4)	28 (59.6)	3 (42.9)	4 (57.1)	67 (46.9)	76 (53.1)
Islam	9 (40.9)	13 (59.1)	7 (38.9)	11 (61.1)	42 (44.7)	52 (55.3)
	χ^2^ = 0.001, p = 0.970		χ^2^ = 0.033, p = 0.997		χ^2^ = 0.108, p = 0.743	
**Marital status**						
Single	27 (40.3)	40 (59.7)	9 (37.5)	15 (62.5)	106 (46.1)	124 (53.9)
Married	1 (50.0)	1 (50.0)	1 (100.0)	0 (0.0)	3 (42.9)	4 (57.1)
	χ^2^ = 0.076, p = 0.783				χ^2^ = 0.029, p = 0.866	
**Level**						
Undergraduates	26 (43.3)	34 (56.7)	10 (40.0)	15 (60.0)	106 (48.0)	115 (52.0)
Postgraduates	2 (22.2)	7 (77.8)	–	–	3 (18.8)	13 (81.3)
	χ^2^ = 1.447, p = 0.229		–	–	χ^2^ = 5.126, p = 0.024^*^	
**Faculty**						
Science and Technology	4 (36.4)	7 (63.6)	0 (0.0)	2 (100.0)	28 (43.8)	36 (56.2)
Agriculture and Forestry	6 (66.7)	3 (33.3)	10 (43.5)	13 (56.5)	34 (45.3)	41 (54.7)
Sociology	4 (50.0)	4 (50.0)	–	–	10 (66.7)	5 (33.3)
Education	4 (26.7)	11 (73.3)	–	–	12 (41.4)	17 (58.6)
Pharmacy	3 (42.9)	4 (57.1)	–	–	11 (68.8)	5 (31.3)
Arts	7 (36.8)	12 (63.2)	–	–	14 (36.8)	24 (63.2)
	χ^2^ = 4.245, p = 0.513		–	–	χ^2^ = 7.859, p = 0.180	
**Institution type**						
University					81 (49.7)	82 (50.3)
College					28 (37.8)	46 (62.2)
					χ^2^ = 2.880, p = 0.090	

*Significant association. Blank cells were courses and levels that were not available in the College study site.

### Factors associated with the perception of TISHIP

Table 4 shows the association between perceptions of TISHIP and the socio-demographic characteristics of respondents. Students numbering 59 (76.6%), aged 19 years and below, had a good perception of TISHIP compared to 59 (69.4%), aged 20–22 years, and 55 (73.3%), aged 23 years and above (Chi-square = 1.072, p = 0.585). Also, 97 (74.6%) students who practised Christianity in the general population had a good perception of TISHIP compared to 76 (80.9%) who practised Islam (Chi-square = 4.877, p = **0.024**). Moreover, 76 (72.4%) university students who practiced Christianity had a good perception of TISHIP compared to 43 (74.1%) who practised Islam (Chi-square = 0.059, p = 0.809). Furthermore, 21 (55.3%) college students who practiced Christianity had a good perception of TISHIP compared to 33 (91.7%) who practiced Islam (Chi-square = 12.422, p = **< 0.001**).

**Table 4:** Association between perception of TISHIP and socio-demographic characteristics.

	University (n = 163)		College (n = 74)		Total (N = 237)	
Variables	Perception of TISHIP		Perception of TISHIP		Perception of TISHIP	
	Good n (%)	Poor n (%)	Good n (%)	Poor n (%)	Good n (%)	Poor n (%)
**Age (Years) (n = 237)**						
≤ 19	30 (75.0)	10 (25.0)	29 (78.4)	8 (21.6)	59 (76.6)	18 (23.4)
20-22	44 (68.8)	20 (31.2)	15 (71.4)	6 (28.6)	59 (69.4)	26 (30.6)
≥ 23	45 (76.3)	14 (23.7)	10 (62.5)	6 (37.5)	55 (73.3)	20 (26.7)
	χ^2^ = 0.988, p = 0.610		χ^2^ = 1.463, p = 0.489		χ^2^ = 1.072, p = 0.585	
**Sex**						
Male	69 (78.4)	19 (21.6)	28 (66.7)	14 (33.3)	97 (74.6)	33 (25.4)
Female	50 (66.7)	25 (33.3)	26 (81.3)	6 (18.7)	76 (71.0)	31 (29.0)
	χ^2^ = 2.833, p = 0.092		χ^2^ = 1.958, p = 0.162		χ^2^ = 0.383, p = 0.536	
**Religion**						
Christianity	76 (72.4)	29 (27.6)	21 (55.3)	17(44.7)	97 (67.8)	46 (32.2)
Islam	43 (74.1)	15 (25.9)	33 (91.7)	3 (8.3)	76 (80.9)	18 (19.1)
	χ^2^ = 0.059, p = 0.809		χ^2^ = 12.422, p = < 0.001*		χ^2^ = 4.877, p = 0.024*	
**Marital status**						
Single	115 (72.3)	44 (27.7)	52 (73.2)	19 (26.8)	167 (72.6)	83 (27.4)
Married	4 (100.0)	0 (0.0)	2 (66.7)	1 (33.3)	6 (85.7)	1 (14.3)
	χ^2^ = 1.516, p = 0.509		χ^2^ = 0.063, p = 0.802		χ^2^ = 0.592, p = 0.442	
**Level**						
Undergraduates	105 (71.4)	42 (28.6)	54 (73.0)	20 (27.0)	159 (71.9)	62 (28.1)
Postgraduates	14 (87.5)	2 (12.5)	–	–	14 (87.5)	2 (12.5)
	χ^2^ = 1.891, p = 0.281		–	–	χ^2^ = 1.831, p = 0.176	
**Faculty**						
Science and Technology	26 (74.3)	9 (25.7)	20 (69.0)	9 (31.0)	46 (71.9)	18 (28.1)
Agriculture and Forestry	22 (73.3)	8 (26.7)	34 (75.6)	11 (24.4)	56 (74.7)	19 (25.3)
Sociology	9 (60.0)	6 (40.0)	–	–	9 (60.0)	6 (40.0)
Education	21 (72.4)	8(27.6)	–	–	21 (72.4)	8 (27.6)
Pharmacy	11 (68.8)	5 (31.2)	–	–	11 (68.8)	5 (31.3)
Arts	30 (78.9)	8 (21.1)	–	–	30 (78.9)	8 (21.1)
	χ^2^ = 2.151, p = 0.828		χ^2^ = 0.388, p = 0.533		χ^2^ = 2.266, p = 0.821	
**Institution type**						
University					119 (73.0)	44 (27.0)
College					54 (73.0)	20 (27.0)
					χ^2^ = 2.153, p = 0.142	

*Significant association

### Predictors of good knowledge of TISHIP among university and college students

Compared to students aged 19 years and below, those aged 20–22 years were less likely to have good knowledge of TISHIP (AOR = 0.896, 95%CI = 0.454 – 1.771, p = 0.753), and those aged 23 years and above were more likely to have good knowledge of TISHIP (AOR = 1.400, 95%CI = 0.669 – 2.932, p = 0.372). Male students were almost as likely as female ones to have good knowledge of TISIP (AOR = 0.943, 95%CI = 0.530 – 1.679, p = 0.842). Students who practiced Christianity were almost as likely as those who practiced Islam to have good knowledge of TISHIP (AOR = 1.145, 95%CI = 0.640 – 2.048, p = 0.648). Also, married students were as likely as their unmarried peers to have good knowledge of TISHIP (AOR = 1.214, 95%CI = 0.214 – 6.878, p = 0.827). Undergraduate students were approximately 10 times more likely to have good knowledge of TISHIP compared to postgraduate students (AOR = 10.489, 95%CI = 2.453 – 44.481, p = **0.002**). University students were three times more likely to have good knowledge of TISHIP compared to college students (AOR = 2.616, 95%CI = 1.209 – 5.658, p = **0.015**) (Table 5).

**Table 5:** Predictors of good knowledge of TISHIP among university and college students.

Variables	Adjusted Odds Ratio	95% Confidence Interval		p-value
		Lower	Upper	
**Age (Years)**				
≤ 19	**1**			
20-22	0.896	0.454	1.771	0.753
≥ 23	1.400	0.669	2.932	0.372
**Sex**				
Male	0.943	0.530	1.679	0.842
Female	**1**			
**Religion**				
Christianity	1.145	0.640	2.048	0.648
Islam	**1**			
**Marital status**				
Single	**1**			
Married	1.214	0.214	6.878	0.827
**Level**				
Undergraduates	10.489	2.453	44.841	0.002*
Postgraduates	**1**			
**Faculty**				
Science and Technology	2.093	0.823	5.326	0.121
Agriculture and Forestry	3.245	1.200	8.776	0.020*
Sociology	3.716	1.021	13.525	0.046*
Education	1.151	0.417	3.180	0.786
Pharmacy	5.997	1.500	23.965	0.011*
Arts	**1**			
**Institution type**				
University	**1**			
College	2.616	1.209	5.658	0.015*

*Significant predictor

### Predictors of good perception of TISHIP among university and college students

Comparing university and college students aged 23 years and above, those aged 19 years and below were twice more likely to have a good perception of TISHIP (AOR = 1.731, 95%CI = 0.759 – 3.949, p = 0.192). Those between the ages of 20 and 22 were as likely as those aged 23 years and above to have good perception of TISHIP (AOR = 1.091, 95%CI = 0.511 – 2.328, p = 0.082). Female students were more likely than males to have good perception of TISHIP (AOR = 1.184, 95%CI = 0.629 – 2.228, p = 0.600). Students who practiced Islam were two times more likely to have good perception of TISHIP than those who practiced (AOR = 2.332, 95%CI = 1.194 – 4.557, p = **0.013**). Married students were two times more likely to have good knowledge of TISHIP compared to their unmarried peers (AOR = 1.839, 95%CI = 0.194 – 17.428, p = 0.594). Postgraduate students were three times more likely to have a good perception of TISHIP compared to undergraduate students (AOR = 3.257, 95%CI = 0.622 – 17.051, p = 0.162). Compared to those in the Faculty of Science and Technology, those in the Faculty of Agriculture and Forestry had nearly equal odds (AOR = 0.983, 95%CI = 0.439 – 2.202, p = 0.967), those in the Faculty of Sociology were 1.7 times less likely (AOR = 0.604, 95%CI = 0.263 – 2.249, p = 0.453), those in the Faculty of Education had nearly equal odds (AOR = 1.322, 95%CI = 0.427 – 4.096, p = 0.628), those studying Pharmacy had nearly equal odds (AOR = 1.247, 95%CI = 0.324 – 4.801, p = 0.748), and those in the Faculty of Arts had two times higher odds of having a good perception of TISHIP (AOR = 1.859, 95%CI = 0.637 – 5.426, p = 0.257). College students were as likely as university students to have good perception of TISHIP (AOR = 1.004, 05%CI = 0.430 – 2.344, p = 0.992) (Table 6).

**Table 6:** Predictors of good perception of TISHIP among both university and college students.

Variables	Adjusted Odds Ratio	95% Confidence Interval		p-value
		Lower	Upper	
**Age (Years)**				
≤ 19	1.731	0.759	3.949	0.192
20-22	1.091	0.511	2.328	0.822
≥ 23	**1**			
**Sex**				
Male	1.184	0.629	2.228	0.600
Female	**1**			
**Religion**				
Christianity	**1**			
Islam	2.332	1.194	4.557	0.013^*^
**Marital status**				
Single	**1**			
Married	1.839	0.194	17.428	0.594
**Level**				
Undergraduates	**1**			
Postgraduates	3.257	0.622	17.051	0.162
**Faculty**				
Science and Technology	**1**			
Agriculture and Forestry	0.983	0.439	2.202	0.967
Sociology	0.604	0.162	2.249	0.453
Education	1.322	0.427	4.096	0.628
Pharmacy	1.247	0.324	4.801	0.748
Arts	1.859	0.637	5.426	0.257
**Institution type**				
University	**1**			
College	1.004	0.430	2.344	0.992

*Significant predictor

## Discussion

TISHIP represents a social security framework that establishes a collaborative relationship between students and the government, operating on the premise of compulsory contributions. These pooled funds are allocated to finance healthcare provisions for students within Nigerian tertiary institutions [7]. The program’s goal is to provide affordable, quality healthcare services to students. However, despite the introduction of the TISHIP by the NHIA to alleviate the out-of-pocket (OOP) health expenditure among tertiary students in Nigeria, OOP payments continue to persist within this population. This could be linked to the level of awareness of the program among students. A key finding of this study revealed that only about half of the students enrolled in the selected tertiary institutions in Ibadan, Oyo State were aware of TISHIP. This low awareness is a major concern, as it can hinder the successful implementation and prevent students from accessing the healthcare services they need.

Several factors contribute to the low awareness of TISHIP among Nigeria tertiary institution students. One major factor is the inadequate communication about the program [17]. Many students were unaware of TISHIP because they had not received sufficient information regarding its benefits, enrolment procedures, and how to access healthcare services [13]. Another factor is the misconception that TISHIP is only accessible to students in public universities. However, TISHIP is available to both public and private tertiary institution students in Nigeria. Unfortunately, less than half of the students had good understanding of the program. A study by Elia et al. in southeastern Nigeria [13] found that only 47.6% of the surveyed students had a good knowledge of TISHIP, highlighting the need for increased awareness of TISHIP among students in Nigeria tertiary institutions. It is essential to educate students on the benefits of the program and how to access healthcare services under it.

Interestingly, respondents who were aware of TISHIP had a positive perception of the program. This suggests that students in Ibadan who knew about TISHIP generally viewed it favourably. The program is designed to provide insurance coverage for students in the event of accidents, illnesses, or other health-related issues, ensuring they have access to healthcare without the burden of high medical bills. A study by Michael et al., in northern Nigeria [18] found that 57% of new graduates (youth corps members) viewed TISHIP as a good initiative. However, this study also revealed that 2.5% of the respondents were reluctant to participate in the program, citing its unreliability, particularly due to frequent drug shortages.

Undergraduate students are more likely to receive continuous and structured information about TISHIP through orientation and matriculation exercises. This study found that undergraduate students below 20 years of age generally had better knowledge of TISHIP compared to postgraduate students. A similar study [6] revealed that undergraduate students were more aware of TISHIP due to their active involvement in campus activities, orientation programs, and health awareness campaigns, which often provide ample opportunities to learn about the program, its benefits, and the enrolment processes [19]. In contrast, postgraduate students, who have more demanding academic and research responsibilities, are less engaged in extracurricular activities and, as a result, have limited access to structured information. Consequently, their knowledge of TISHIP may be limited to informal discussions or incidental exposure.

However, postgraduate students had a more positive perception of TISHIP compared to undergraduates. This could be due to their higher level of exposure to information and resources, as they are typically more engaged in research, attend conferences, and have greater access to academic networks, which could lead to a better understanding of TISHIP and its benefits. Additionally, postgraduate students may face more demanding academic and research requirements, which can increase stress levels and health concerns. This might make them more appreciative of the benefits offered by TISHIP.

This study also revealed that married students generally had a better perception of TISHIP compared to their unmarried counterparts. This finding aligns with previous research [19–21] that has emphasized the positive influence of marital status on individuals’ access to and perception of healthcare services. Married individuals tend to seek medical assistance more promptly and are more likely to take advantage of preventive measures and regular check-ups [22].

The positive perception of TISHIP among students below 20 years of age can be attributed to several factors. Health insurance is more prevalent and widely accepted in this age group, and younger students are more likely to have been exposed to the importance of health insurance from an early age. Additionally, they may have fewer pre-existing health conditions, making them less sceptical about the benefits of TISHIP.

The findings of this study are consistent with existing literature [23,24] on health insurance awareness and education among different educational cohorts. Several studies [25–28] have observed similar patterns, emphasizing the role of age, educational level, and institutional support in determining health insurance knowledge. However, our findings contrast with some international studies [29,30]. For example, a study by Hargreaves et al. conducted in 11 high-income countries (Australia, Canada, France, Germany, the Netherlands, New Zealand, Norway, Sweden, Switzerland, United Kingdom, United States) found that older adults had a more favourable perception of health insurance compared to younger individuals. This difference may be attributed to variations in healthcare systems, cultural norms, and the overall perception of insurance in various countries. One possible explanation for the differing perceptions between older and younger students in the Nigerian context could be the level of exposure to information about TISHIP. Younger students in Nigeria are more actively engaged with technological platforms, social media, and online resources [31], providing them with more information about the benefits and functioning of health insurance. In contrast, older students may rely more on traditional sources of information, which may be less accessible or comprehensive.

## Limitation

One limitation of this study is the potential impact of sample size and variability on the statistical analysis. This variability may limit the generalizability of some findings. We acknowledge this limitation and suggest that future studies with larger sample sizes may provide more robust conclusions.

## Conclusion

TISHIP is a significant step towards ensuring that tertiary students in Nigerian tertiary institutions have access to quality healthcare services. By providing affordable healthcare, it aims to improve the health and wellbeing of students. However, the low awareness of TISHIP among these students remains a major concern. To address this, it is important to implement more extensive awareness campaigns about the program. The government and relevant stakeholders must actively promote TISHIP to increase enrolment and ensure that students are informed about its benefits and how to access healthcare services. Efforts should be made to continuously enhance communication about the program to ensure that students can fully benefit from the healthcare services under TISHIP.

## Recommendations

Schools and other stakeholders must proactively educate students about TISHIP’s benefits and its enrolment procedures. This can be achieved through various means, like the use of information and communication technologies (ICTs) such as social media and mobile applications, health promotion campaigns, and seminars.
